# Morphologic, molecular and metabolic characterization of *Aspergillus* section *Flavi* in spices marketed in Lebanon

**DOI:** 10.1038/s41598-019-41704-1

**Published:** 2019-03-27

**Authors:** Joya Makhlouf, Amaranta Carvajal-Campos, Arlette Querin, Soraya Tadrist, Olivier Puel, Sophie Lorber, Isabelle P. Oswald, Monzer Hamze, Jean-Denis Bailly, Sylviane Bailly

**Affiliations:** 1 Toxalim (Research Center in Food Toxicology), Université de Toulouse, INRA, ENVT, INP-Purpan, UPS, 180 Chemin de Tournefeuille, F-31027 Toulouse, France; 2 Health and Environment Microbiology Laboratory, Lebanese University, Beirut, Lebanon

## Abstract

Spices are used extensively in Lebanon not only to flavour foods but also for their medicinal properties. To date, no data are available regarding the nature of the toxigenic fungal species that may contaminate these products at the marketing stage in this country. Eighty samples corresponding to 14 different types of spices were collected throughout Lebanon to characterize the *Aspergillus* section *Flavi* contaminating spices marketed in Lebanon and the toxigenic potential of these fungal species. Most fungal genera and species were identified as belonging to *Aspergillus* section *Flavi*. *Aspergillus flavus* was the most frequent species, representing almost 80% of the isolates. Although identified as *A. flavus* by molecular analysis, some strains displayed atypical morphological features. Seven strains of *A. tamarii* and one *A. minisclerotigenes* were also isolated. Analyses of toxigenic potential demonstrated that almost 80% of strains were able to produce mycotoxins, 47% produced aflatoxins, and 72% produced cyclopiazonic acid, alone or in combination with aflatoxins.

## Introduction

In Lebanon, as in many Mediterranean countries, spices are used extensively not only to flavour foods but also for their medicinal properties. However, spices can be contaminated with various hazards^1–3^, among which toxigenic fungi are probably the most important. Indeed, some fungal species produce toxic secondary metabolites named mycotoxins as they develop on human food and animal feed^4^. Among the hundreds of known mycotoxins, aflatoxins are the major ones for public health because they are the most potent of the known natural carcinogens, and the International Agency for Research on Cancer classified aflatoxin B1 (AFB1) in the group of molecules that are carcinogenic for both humans and animals (group 1)^5^. Chronic exposure to AFB1 is a major cause of hepatocarcinoma^6^ and this food contaminant has been associated with the highest number of DALYs (deaths and disability adjusted life years)^7^. Aflatoxins may contaminate many foods including cereals, dry fruits, and groundnuts. They are also frequent contaminants of spices. Indeed, spices are mainly produced in areas where both temperature and humidity favour fungal development and subsequent toxinogenesis. Methods of post-harvest processing (sun drying, handling, storage) can also allow secondary contamination and the development of moulds^8^. Previous studies have demonstrated that spices can be contaminated by mycotoxins and thus represent a direct source of exposure for consumers^9–11^, as recapitulated in a recent review^12^. That is why spices are specifically concerned by regulations on aflatoxins. For instance, the E.U. regulation restricts contamination to 10 µg/kg for total aflatoxins and 5 µg/kg for AFB1^13^. However, contamination of spices may exceed regulations and justify the withdrawal of contaminated products. As an illustration, in 2016, the European Rapid Alert System for Food and Feed recorded 79 notifications of mycotoxin contamination of spices and herbs, most of which corresponded to the presence of aflatoxin B1 at levels exceeding European limits^12,14^.

**Figure 1:**
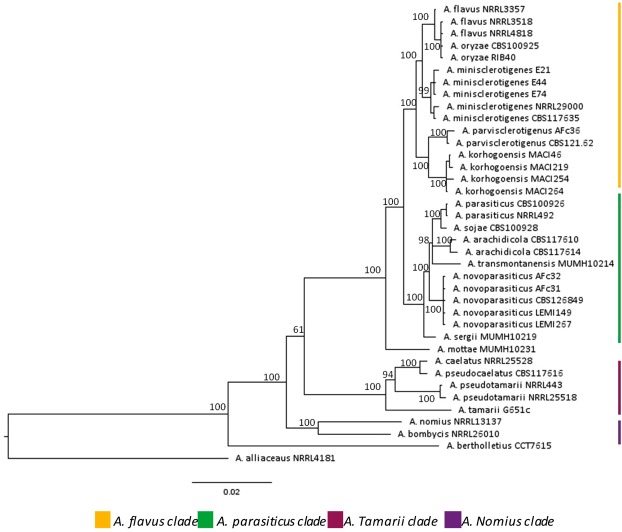
Phylogenetic tree of *Aspergillus* section *Flavi*. The phylogenic tree is based on concatenated sequences from 4 genomic loci (*benA*, *cmdA*, *PreB* and *rpb1*). Bayesian tree was calculated from 38 strains and includes the type strains for most species. Strong bootstraps are shown at branch nodes. The sequences of primers and protocols used to generate gene fragments are available in Carvajal-Campos *et al*.^19^. *Petromyces alliaceus* NRRL 4181 was used as an outgroup taxon. (Adapted from Carvajal-Campos^51^).

Aflatoxins are produced by different fungal species that belong to the genus *Aspergillus* and more specifically to the *Flavi* section. For years, three main aflatoxigenic species were commonly considered in the section *Flavi*: *A. flavus*, *A. parasiticus* and *A. nomius*^15^. In the last decade, the use of molecular tools enabled the identification of new species belonging to the section *Flavi*, comprising 34 different species, of which 17 are aflatoxigenic^16–22^. These species can be distinguished by subtle morphological specificities, molecular changes in some gene sequences^22^ (Fig. 1) and, most importantly, through their ability to produce different mycotoxins^16,23^. Indeed, some species, including *A. flavus*, *A. pseudotamarii* and *A. togoensis*, produce aflatoxins of B type, whereas others, including *A. parasiticus, A. minisclerotigenes, A. mottae, A. nomius*, *A. novoparasiticus, A. arachidicola* and *A. korhogoensis* produce both B and G type aflatoxins^16,23^. Some species may also produce other toxic secondary metabolites such as cyclopiazonic acid (CPA)^24^.

To date, only few studies aimed to characterized the nature of fungi and more precisely of *Aspergillus* section *Flavi* that may contaminate spices (Table 1). In most cases, fungal identification was done using a morphologic approach which may not have allowed the discrimination of recently identified species among the *Flavi* section. On the same way, the ability to produce aflatoxins was usually done with no distinction whether they belong to B or G type. With one exception^9^, the production of cyclopiazonic acid was not tested. No data are available regarding the nature of the fungal species that may contaminate spices at the marketing stage in Lebanon despite such knowledge is extremely important for risk assessment.

**Table 1:** Fungal contamination of spices and toxigenic potential of isolates.

Country	Type of spices	Number of samples	Method used for fungal identification	Incidence of *Aspergillus flavus* (%)	Toxigenic potential (nature of toxin tested)	Incidence of toxigenic strains	Reference
Brazil	Rosemary, cinnamon, clove, fennel, pepper, pepperoni, oregano	200	Morphology	15	Aflatoxins^a^	38%	^26^
India	Red chili, black pepper, turmeric, coriander, cumin, fennel, caraway, fenugreek, ginger	311	Morphology	19.3	Aflatoxins	20–56%^b^	^27^
Morocco	Paprika, cumin, black pepper, white pepper	80	Morphology and gene sequence	78	Aflatoxin B1, B2, G1, G2 and CPA	57%	^9^
Saudi Arabia	Cinnamon, cumin, sumac, ginger, saffron, fenugreek, pepper, fennel, thyme, cardamom, caraway, aniseed, clove	138	Morphology	17.4	ND	ND	^28^

^a^no distinction between B and G aflatoxins.

^b^Incidence of toxigenic strains vary according to spice samples.

ND: not determined.

Within this context, the aim of this study was to finely characterize the *Aspergillus* section *Flavi* that can contaminate spices marketed in Lebanon and to determine the toxigenic potential of the isolated strains.

## Results

### Fungal counts and identification

**Figure 2:**
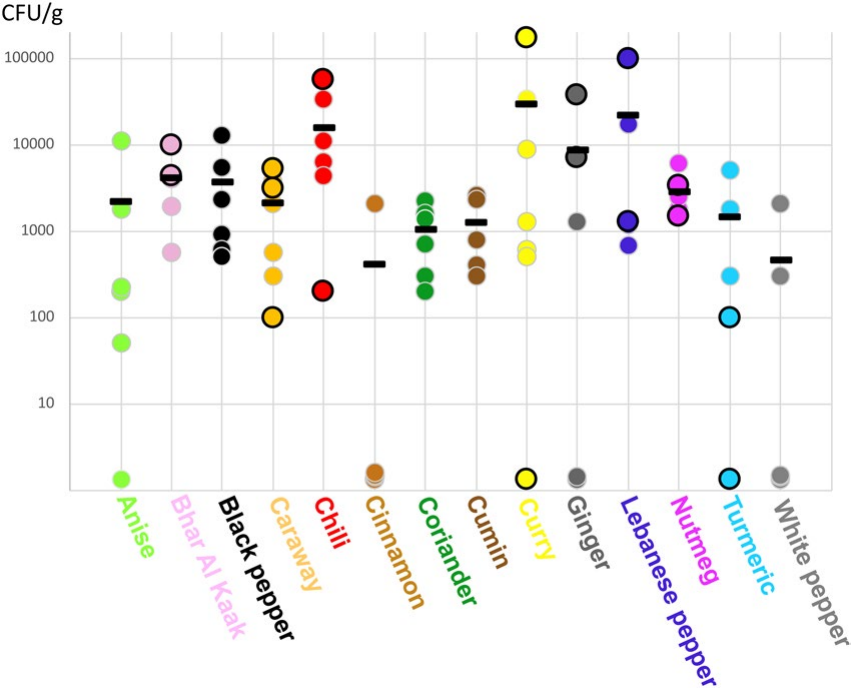
Total fungal load of spices samples (CFU/g). Fungal CFU were counted after 3 and confirmed after 5 days of culture on both MEA and salted MEA at 25 °C. Dots represent total fungal load of each sample whereas short bold lines represent the mean fungal load of each kind of spice. Dots circled in black correspond to packaged samples.

The total fungal counts of the spice samples are presented in Fig. 2. The overall mean fungal contamination was 7.6 × 10^3^ CFU/g, with marked variations among the samples. Most spices displayed a moderate mean fungal load that ranged from 10^3^ to 4 × 10^3^ CFU/g. These loads were associated with spices harvested as seeds or dried grains. Some samples were even less contaminated. For instance, three out of five white pepper samples displayed no detectable fungal contamination. Four out of five cinnamon samples were found to be uncontaminated. Some ginger and turmeric samples were also found to be uncontaminated, but in smaller percentages (2/5 and 1/5 respectively).

Other spices were heavily contaminated with fungi. This was the case for spices resulting from a mixture of several ingredients such as Baar El Kaak, curry and Lebanese pepper. Heavy contamination was also the case for chili samples, which displayed a mean level of contamination of 2 × 10^4^ CFU/g.

Packaging did not limit fungal contamination of the spices. Indeed, for six out of the eight spices for which conditioned samples were analysed (caraway, chili, curry, Lebanese pepper, ginger, and Bhar Al Kaak), the most contaminated samples were the conditioned ones.

Identification of the fungal genera present in spices showed that *Aspergillus* was the most frequent genus, with *Aspergillus* isolates observed in 76% of samples (Table 2). *Aspergillus* was followed, in decreasing order, by Mucorales (36%), *Penicillium* (30%) and *Fusarium* (7.5%). The phytopathogenic genus *Fusarium* was only found in spices prepared from rhizomes (turmeric and ginger) or fruits (chili). Other fungal genera such as *Cladosporium, Alternaria, Acremonium*, and *Verticillium*, which mostly are derived from field flora, as well as *Paecilomyces, Spicaria* and *Hemispora*, were also present at lower levels. The complete analysis of fungal contamination of each sample is reported in Supplementary Table 1 (Table S1).

**Table 2:** Fungal genera present in spice samples.

Fungal genus Spice	*Aspergillus*	Mucorales	*Penicillium*	*Fusarium*	Other
*Anise*	4/6	2/6	2/6	1/6	3/6
*Bhar al Kaak*	5/5	4/5	4/5	0/5	1/5
*Black pepper*	6/6	2/6	3/6	0/6	4/6
*Caraway*	7/7	5/7	2/7	0/7	5/7
*Chili*	5/7	2/7	1/7	2/7	1/7
*Cinnamon*	0/5	0/5	1/5	0/5	1/5
*Coriander*	6/6	2/6	1/6	0/6	2/6
*Cumin*	4/5	2/5	2/5	1/5	4/5
*Curry*	6/7	5/7	0/7	0/7	2/7
*Ginger*	3/5	0/5	0/5	1/5	2/5
*Lebanese pepper*	5/5	3/5	4/5	0/5	2/5
*Nutmeg*	6/6	1/6	1/6	0/6	3/6
*Turmeric*	2/5	2/5	1/5	1/5	1/5
*White pepper*	2/5	2/5	0/5	0/5	0/5
**Total**	**61/80**	**29/80**	**24/80**	**6/80**	**31/80**

Results are expressed as the number of contaminated samples.

**Figure 3:**
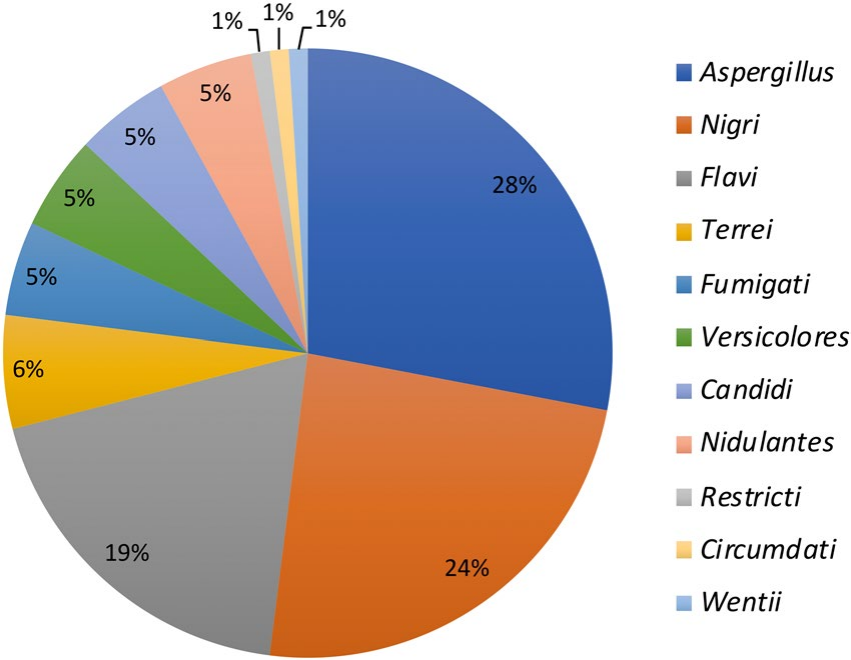
Relative proportion of *Aspergillus* sections in spice samples. Isolates belonging to *Aspergillus* genus were identified at the section level by both macroscopic and microscopic examination after five and seven days of culture at 25 °C on MEA, according to Pitt and Hocking^45^ and Samson *et al*.^46^. The relative proportion of each section was then calculated based on the total number of isolates from the 80 samples analysed.

*Aspergillus* isolates belonged to 11 sections (Fig. 3), among which three predominated. Indeed, *Aspergillus* section *Aspergillus* isolates were found in almost 60% of the samples. They were present in all kinds of spices with the exception of cinnamon. Isolates of the *Nigri* section were also very common and were found in almost 50% of the analysed samples but not in the cinnamon or ginger. The section *Flavi* was observed in 40% of the samples. The distribution of these isolates was similar to that observed for those from the *Nigri* section. Isolates belonging to five other sections (*Fumigati, Terrei, Versicolores, Candidi* and *Nidulantes*) were also identified in 10 to 12% of the samples. Isolates belonging to the *Restricti* section were found in one sample of curry and one sample of nutmeg. In the same line, three isolates belonging to section *Circumdati* (2) and *Wentii* (1) were found in Lebanese pepper (2/3) and Bhar Al Kaak samples (1/3), respectively. Ten out of 11 sections were observed in Lebanese pepper.

### Characterization of *Aspergillus* section *Flavi*

Strains of *Aspergillus* section *Flavi* were heterogeneously distributed in 40% of the samples. They were present in all Bhar Al Kaak (5/5) samples and in most chili (5/7) and curry (4/7) samples. By contrast, they were only isolated in one sample of caraway, cumin, white pepper, and anise and in two samples of curcuma and coriander. No isolates of *Aspergillus* section *Flavi* were found in either cinnamon or ginger samples. A total of 53 strains were isolated, with some samples displaying several morphologically distinct isolates that were purified and further identified. For instance, 12 strains were obtained from five out of the seven samples of chili analysed.

#### Identification of *Aspergillus* species

Both morphological and molecular identification were performed to identify isolates at the species level. To this end, strains were cultivated on Malt Extract Agar (MEA), CREatinin sucrose Agar (CREA) and *Aspergillus flavus* and *parasiticus* Agar (AFPA) media, and their macroscopic and microscopic features were analysed. In parallel, ITS, *benA* and *cmdA* genes were sequenced. Complete results are given in Supplementary Table 2 (Table S2).

Forty-five out of the 53 isolates (85%) were identified as *A. flavus* isolates by molecular analysis. Among them, 42 displayed typical *A. flavus* morphological features, with a yellowish green colony, colourless or yellowish on the reverse side and long conidiophores that are rough in the distal part, with mostly radiate and biseriate heads and with some columns in aerial mycelium. Conidia are oblong and mildly rough. Some variations occurred in the depth and colour of the colony and sclerotia formation (Table S2). For instance, the J33a, J34a, J42a and J62a isolates displayed dark olive-green colonies with numerous uniseriate columnar *Aspergillus* heads. The J117a and J86a isolates produced large dispersed sclerotia, whose diameter exceeded 500 nm, whereas only the J7 isolate produced numerous small sclerotia (approximately 500 nm in diameter). All typical *A. flavus* isolates produced aspergillic acid, as evidenced by the presence of a bright orange reverse on the AFPA medium. The production of organic acid that resulted in yellow discoloration of the CREA medium varied with the isolate (Table S2).

**Figure 4:**
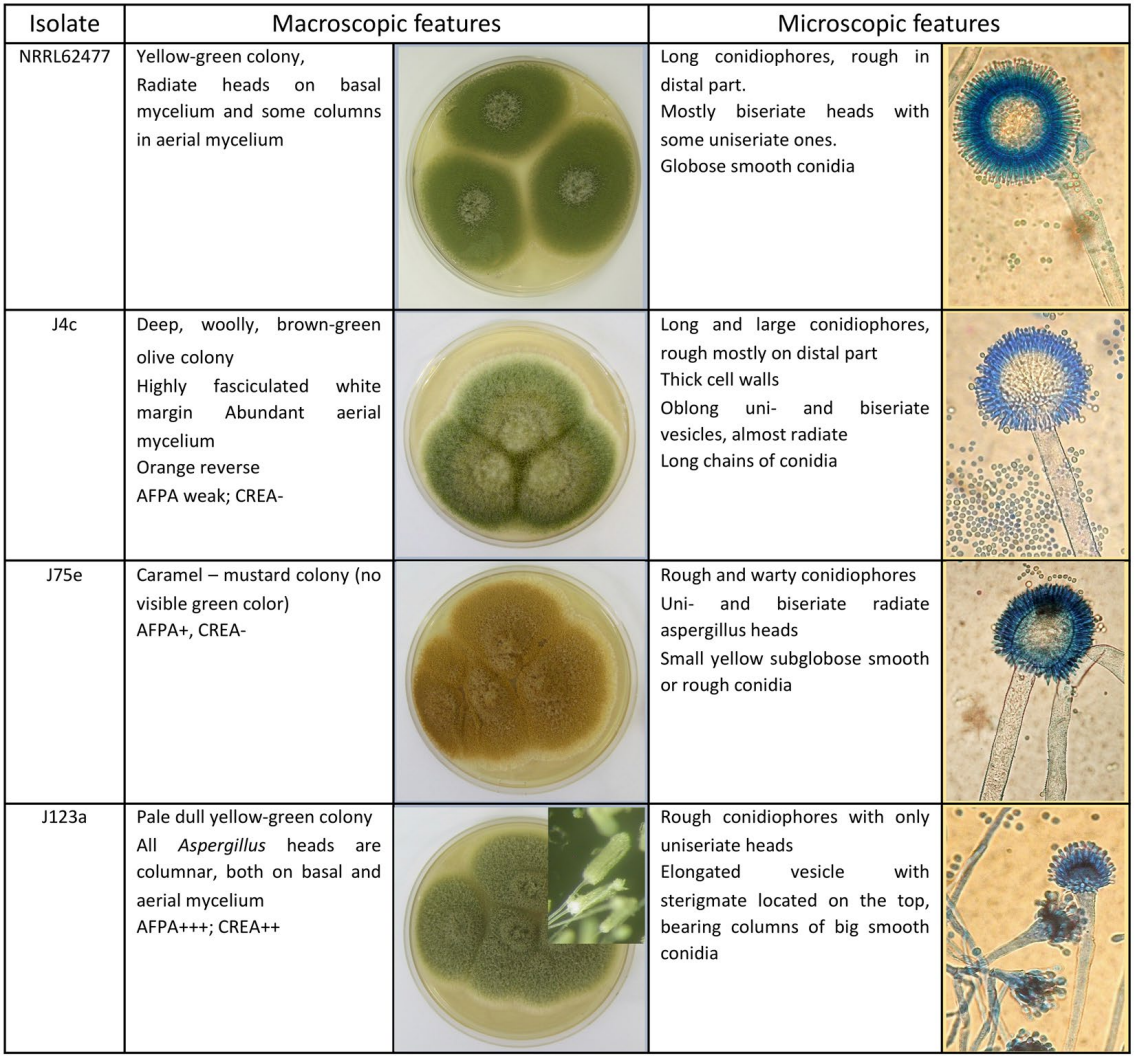
Main macro- and microscopic features of atypical *A. flavus* isolates compared to the referenced *A. flavus* NRRL62477 after 7 days of culture on MEA at 25 °C. Some isolates of the *Aspergillus* section *Flavi* displayed unusual morphologic features but were nevertheless identified as *Aspergillus flavus* by molecular identification (sequence of β-tubulin and calmodulin genes) (Table 6). Compared to a reference strain (NRRL 62477, previously isolated from paprika^9^) displaying typical *Aspergillus flavus* features, those atypical strains mainly differ by colour and aspect of conidial heads. The presence of such isolates demonstrates the strong phenotypical diversity that may exist within *Aspergillus flavus* species.

Three isolates displayed unusual morphological features but were nevertheless identified as *A. flavus* by molecular analysis; these findings are summarized in Fig. 4.

Seven strains (J4a, J6a, J31a, J37, J63c, J116b, and J118a) were identified as *A. tamarii* by morphological and molecular analyses. These strains were isolated from different spices including chili (2 isolates), black pepper (1), white pepper (1), Lebanese pepper (1), curry (1) and Bhar al Kaak (1). They displayed highly sporulated dark green-bronze colonies. The conidiophores were long, wide and finely rough. The vesicles were oblong, bearing divergent chains of big, round echinulate spores. All *A. tamarii* strains displayed a dark-brown reverse side on the AFPA medium.

One isolate (J117c) was identified as *A. minisclerotigenes*. This strain was obtained from a sample of Bhar al Kaak. It produced numerous small sclerotia with diameters <400 nm.

#### Toxigenic potential of *Aspergillus* section *Flavi* strains

Since the section *Flavi* contains species with differing abilities to produce mycotoxins, we further characterized the ability of the strains to produce different toxins. All *Flavi* section strains were analysed for the production of both aflatoxins of B and G type as well as CPA. Details on the levels of production of the different compounds are listed in Table S2.

Nearly half of the isolates (25/53) produced AFB1. Many isolates produced CPA alone (19/53) or simultaneously with aflatoxin (19/53). A total of 83% of the isolates were able to produce at least one toxin (Table 3).

**Table 3:** Chemotypes of *Aspergillus* section *Flavi* isolated from spices.

Chemotype Spice	Number of isolates	Chemotype I (AFB+/CPA+)	Chemotype II (AFB+/AFG+/CPA+)	Chemotype III (AFB+)	Chemotype IV (CPA+)	Chemotype V (no toxin)
Anise	2	—	—	—	2/2	—
Bhar al Kaak	8	4/8	1/8	—	3/8	—
Black pepper	7	2/7	—	1/7	2/7	2/7
Caraway	1	—	—	—	—	1/1
Chili	12	5/12	—	2/12	4/12	1/12
Cinnamon	0	—	—	—	—	—
Coriander	3	—	—	—	3/3	—
Cumin	1	—	—	—	—	1/1
Curry	6	3/6	—	—	1/6	2/6
Ginger	0	—	—	—	—	—
Lebanese pepper	3	1/3	—	—	2/3	—
Nutmeg	3	1/3	—	1/3	—	1/3
Turmeric	6	2/6	—	2/6	1/6	1/6
White pepper	1	—	—	—	1/1	—
**Total (%)**	**53**	**18/53 (34%)**	**1/53 (2%)**	**6/53 (11%)**	**19/53 (36%)**	**9/53 (17%)**

Eighty percent of *A. flavus* strains were able to produce mycotoxins, of which 40% (18/45) produced both AFB and CPA (Chemotype I), whereas 27% (12/45) produced only CPA (Chemotype IV), and 13% (6/45) produced only AFB (Chemotype III). As expected, none of the *A. flavus* strains produced AFG (Chemotype II). All *A. tamarii* strains produced only CPA, and the *A. minisclerotigenes* strain produced both B and G aflatoxins, as well as CPA.

When the toxigenic potential of strains is considered as a function of the nature of the spice from which they were isolated, approximately 60% of the strains isolated from chili, Bhar al Kaak and curcuma produced AFB1. By contrast, none of the few isolates from anise, white pepper, caraway, cumin, and coriander were aflatoxigenic.

Some strains displayed a high toxigenic potential, producing more than 200 µg aflatoxins/Petri dish after 7 days at 25 °C. To better evaluate the risk associated with the presence of such strains in spices, the ratio of AF/CFU was calculated. As shown in Table 4, if a strain with high toxigenic potential contaminated a spice sample, 2 to 6 × 10^4^ CFU/g could be sufficient to reach the E.U. regulation of 10 ng aflatoxins/g. Fortunately, in most cases, the fungal contamination levels observed for these strains in analysed samples were much lower, ranging from a few tens to a few hundred CFU/g. However, for one sample, a contamination level of 10^5^ CFU *Aspergillus flavus*/g was measured, demonstrating the possibility for reaching and exceeding the threshold, especially in cases of non-optimal storage.

**Table 4:** Estimation of the risk associated with the presence of highly aflatoxigenic strains in spices.

Strain	Aflatoxin production (µg/Petri dish)^a^	CPA production (µg/Petri dish)^a^	CFU (/Petri dish ± SD)^a^	AF/CFU ratio (×10^7^)	Theoretical number of CFU to reach 10 ng µg AF^b^ (×10^4^)	Number of CFU/g observed in samples
J4b	213	376	1.13 × 10^9^ ± 9.4 × 10^7^	1.83	5.46	50
J6b	318	199	1.2 × 10^9^ ± 2 × 10^8^	2.65	**3.77**	**100,000**
J71a	251	333	1.49 × 10^9^ ± 2.2 × 10^8^	1.69	5.91	1,000
J75c	351	127	1.16 × 10^9^ ± 4 × 10^7^	3.03	3.30	200
J86a	578	127	1.2 × 10^9^ ± 2 × 10^8^	4.28	2.07	2,000
J117b	336	4532	1.48 × 10^9^ ± 1.4 × 10^8^	2.27	4.41	150

^a^after 7 days at 25 °C.

^b^E.U. regulation on AF content in spice is set at 10 ng/g.

## Discussion

The aim of this study was to investigate the presence of *Aspergillus* section *Flavi* in spices marketed in Lebanon. To this end, 80 samples of commonly used spices were collected in markets, and the fungal contamination was analysed. The average fungal load of samples agreed with that found in other recent surveys conducted in other countries^25,26^. Some differences were observed between spices, which could be due to either the composition of the condiments or to the preparation process. For instance, some spices, such as cinnamon or anise, are rich in phenolic compounds that may have antifungal properties, which may explain their lower fungal contamination^27,28^. Similarly, the terpenes in ginger and turmeric and an unidentified protein in ginger were shown to have antifungal activity, which may help explain the low level of fungal contamination of these spices^29^.

In the case of other spices, the preparation process may also help reduce the initial fungal load by removing the external parts of the berries/roots before marketing. For instance, white pepper, which is obtained by removing the pericarp from the berries, was less contaminated than black pepper. In the same way, spices obtained from roots that are peeled during their preparation (i.e., ginger and turmeric) were less contaminated than spices obtained from whole dried seeds or fruits. Spices prepared by mixing different plants/seeds were usually more contaminated than others, as observed for Bhar Al Kaak (a mixture of black pepper, fennel, cardamom and clove), curry (mixture of ginger, garlic, onion, coriander, cinnamon) and Lebanese pepper (mixture of black pepper, chili, coriander and cinnamon). Each ingredient used in these preparations represents a separate source of contamination; moreover, the mixture of these ingredients may be an additional source of contamination. Since each ingredient may also display variable water activity, the water activity of the final mixture is unstable, and finding the appropriate storage procedure is complex. As a demonstration, these complex samples were the ones most frequently contaminated with mucorales, which are hygroscopic fungi.

In this study, we also analysed the fungal load of samples that were packed in hermetically sealed plastic bags for sale. Surprisingly, this did not prevent fungal contamination; instead, for six out of the eight types of conditioned spices that we analysed, the most highly contaminated samples were the conditioned ones. Therefore, it appears that the traditional spice storage procedure, using open plastic containers that allow ventilation and limiting water migration and condensation, should be preferred to hermetically sealed packaging, which should be reserved for completely dried or sterilized spices. The development of breathable materials, allowing air to pass and avoiding condensation but also protecting against external contaminations, would probably be of interest to improve the preservation of spices.

*Aspergillus* was the main genus found in the samples, and although a great variety of sections were present, sections *Nigri* and *Flavi* were very frequent, which is also in agreement with data reported in Morocco, India or Brasil^9,30–32^. *Aspergillus* section *Aspergillus*, formerly named *Eurotium*^33^, which corresponds to xerophilic isolates, was also frequently observed, as expected in dry commodities.

Fifty-three distinct strains of *Aspergillus* section *Flavi* were isolated from samples, among which 85% were identified as *A. flavus* by molecular analysis, demonstrating relatively low biodiversity within the section *Flavi*. Although the molecular identification agreed with the results of morphological examination of the isolates in most cases, a few isolates displayed atypical morphological features even though they were identified as *A. flavus* strains by molecular analysis. In the last decade, the use of molecular tools and gene sequencing has shown that the *Flavi* section is much more complicated than previously thought, and many new species have been introduced, increasing the number to 34^21,22^. From our results, it appears that, beyond this genetic diversity, isolates identified as *A. flavus* may also present some morphological particularities, demonstrating the wide phenotypic variation within the species. Two of the three strains with unusual morphology were able to produce mycotoxins (AFB and CPA), demonstrating the importance of these isolates that could easily have been misidentified due to their atypical colouration. Since these particular isolates originated from different spices, this phenotypic variety does not appear to be correlated with substrate specificity but could rely on the presence of mutations on genes involved in pigment synthesis or on a distinct basal expression of these genes^34,35^.

Seven out of 53 isolates (13%) were identified as *A. tamarii*. This proportion agrees with the results of previous studies reporting the presence of *A. tamarii* in spices, dry fruits and cereals^25,30,36–38^. As expected, all these isolates produced CPA^24^.

Finally, only one isolate was identified as *A. minisclerotigenes*. This species was first described in 2008^23^. It has been reported in several geographic areas and on different substrates such as groundnuts in Argentina^23^, groundnuts in Algeria^39^, maize and almonds in Portugal^17^ and in spices marketed in Morocco^9^. *Aspergillus minisclerotigenes* is of interest due to its ability to produce both B and G aflatoxins together with CPA. However, this species is also quite difficult to isolate due to weak sporulation when cultured on MEA.

Our results show that more than 80% of the isolates belonging to the section *Flavi* are toxigenic. If we consider aflatoxin production, 47% of the strains produced AFB1 (chemotypes I and III). This proportion of aflatoxigenic strains agrees with data available on the toxigenic potential of *Aspergillus* section *Flavi* in different foodstuffs such as spices, chestnuts and cereals^9,31,38,40^. Some strains were able to produce great amounts of aflatoxins when cultured *in vitro*. The presence of such highly toxigenic strains raise the question of the sanitary impact of the contamination. From data obtained while characterizing toxigenic potential, we demonstrated that the presence of only a few thousands CFU of these strains could be sufficient to reach the regulatory limit on the aflatoxin content in spices. Even if most samples of our study displayed relatively low levels of contamination, such fungal counts can be obtained, as observed in one sample of this study. This underscores the need for proper storage procedures, especially for spices that result from a mixture of different ingredients. The use of conditioning materials that protect from contamination but that also enable ventilation could improve the preservation of spices and limit the risk of fungal development and subsequent contamination with aflatoxins.

Notably, a high proportion of the strains were able to produce CPA, alone or in combination with aflatoxins. Although this mycotoxin has already been reported as a possible contaminant of several foods and feeds^41^, recent data are lacking on the contamination of foods and feeds with that CPA-producing species. Notably, the production of CPA by the *A. flavus* strains used as biocontrol agents appeared to be a strong limitation, which needs to be evaluated since CPA has been demonstrated to be cytotoxic and immunotoxic to human cells^42,43^. Moreover, due to its frequent coproduction with aflatoxins, an investigation of the possible interaction between the two toxic compounds when ingested simultaneously would be interesting. Considering the origin of toxigenic strains, we observed that some spices are more often contaminated with toxigenic isolates than others. For example, 58, 62 and 67% of the strains from chili, Bhar al Kaak and turmeric, respectively, are toxigenic. By contrast, none of the few strains of anise, black pepper, caraway, coriander and cumin were shown to be toxigenic. This finding is also of interest regarding the risk assessment of contamination of these spices with mycotoxins.

In conclusion, this study reports, for the first time, the diversity of *Aspergillus* section *Flavi* in spices marketed in Lebanon. The results reveal that *A. flavus* is the most frequent species in the samples analysed and that the proportion of toxigenic strains is high. This study also demonstrated that some spices are frequently contaminated with toxigenic strains and should probably be submitted to specific safety controls.

## Methods

### Solvents and reagents

All solvents were of HPLC grade and purchased from VWR International (Fontenay-sous-Bois, France). Medium and agar were purchased from BIOKAR Diagnostics (Allonne, France), and Tween 80, from Merck (Darmstadt, Germany). Mycotoxin standards were purchased from Sigma (Saint-Quentin-Fallavier, France). Aflatoxins were dissolved in toluene-acetonitrile (98:2, v/v) and cyclopiazonic acid in methanol to obtain 200 µg/ml stock solutions that were stored at −20 °C. Standard solutions used for HPLC analysis were stored at 4 °C and changed monthly. Taq DNA polymerase used for molecular identification was purchased from Invitrogen (Carlsbad, Ca, USA).

### Samples

Eighty samples were randomly collected from several retailers across all Lebanese regions. Fourteen different types of spices were analysed: anise (n = 6), Bhar Al Kaak (n = 5), caraway (n = 7), chili (n = 7), cinnamon (n = 5), coriander (n = 6), cumin (n = 5), curry (n = 7), ginger (n = 5), nutmeg (n = 6), black pepper (n = 6), white pepper (n = 5), Lebanese pepper (n = 5), turmeric (n = 5). All samples were of commercial grade, and to be representative of what can enter human food chain, no macroscopically mouldy sample was taken.

Sixty-three out of the 80 spices samples were taken from batches stored in plastic containers with no particular protection. Some samples were hermetically sealed in plastic bags before marketing (17 samples including Bhar Al Kaak (n = 2), caraway (n = 3), chili (n = 2), curry (n = 2), ginger (n = 2), nutmeg (n = 2), Lebanese pepper (n = 2) and turmeric (n = 2).

Approximately 100 g of each sample were collected and carefully placed in 200 ml sterile plastic jars (Thermo-Fischer, Dardilly, France). To avoid any fungal development without altering spore viability, samples were stored at 4 °C until analysis^9,44^. In the laboratory, samples in the form of grains were finely ground in a Waring blender (Waring Laboratory, Torrington, CT, USA).

### Fungal count and identification

Twenty grams of each sample were mixed with 180 ml of 0.05% Tween 80 for 2 min in a Waring blender and then placed on a horizontal shaking table at 220 rpm for one hour. Decimal dilutions were prepared in 0.05% Tween 80, and 100 µl of each dilution were plated on both MEA medium and Salted MEA (MEA + 6% NaCl)^9^. This last medium was used to identify xerophilic species and to limit Mucorale development that may prevent a correct enumeration and identification of species with a low growth rate. Fungal colonies were counted after three days of culture at 25 °C and confirmed after five days. The limit of detection for the fungal count was 10 CFU/g of sample. The colonies were then identified according to Pitt and Hocking^45^ and Samson *et al*.^46^. *Aspergillus* section *Flavi* strains were isolated from plates by several platings on MEA and Salted MEA.

### Characterization of *Aspergillus* section *Flavi* strains

#### Morphological identification

*Aspergillus* section *Flavi* strains were identified at the species level by both macroscopic and microscopic examination after five and seven days of culture at 25 °C on MEA, CREA (3.0 g creatine, 30 g sucrose, 0.5 g KCl, 0.5 g MgSO_4_.7H_2_O, 0.5 g FeSO_4_.7H_2_O, 1.3 g K_2_HPO_4_.3H_2_O, 0.05 g Bromocresol purple, 15.0 g agar, distilled water to bring to 1 litre) and AFPA (Thermo-Fisher Diagnostics, Dardilly, France) media according to Pitt *et al*.^47^ and Varga *et al*.^16^.

#### Molecular identification

The molecular identification of all *Aspergillus* section *Flavi* strains was first performed by internal transcribed spacer (ITS) rRNA sequencing. For strains displaying atypical morphological features, this was completed by beta-tubulin (*benA*) and calmodulin (*cmdA*) gene amplification and sequencing. The strains were cultured in yeast extract sucrose (YES) broth and placed on a shaking incubator at 160 rpm at 27 °C for three days. Genomic DNA was isolated from mycelia, as previously described^48^. Primers used for the molecular identification are listed in Table 5. PCR reactions were carried out in a GeneAmp PCR 2700 thermocycler (Applied Biosystems, Foster City, USA). PCR products were purified with a GenElute PCR Clean-Up Kit (Sigma-Aldrich) and sequenced on an ABI3130XL sequencer (Applied Biosystems) using the dye terminator technology. PCR products were sequenced in both directions. Nucleotide sequence accession numbers for *benA* and *cmdA* from *A. flavus* with atypical morphology, *A. tamarii* and *A. minisclerotigenes* are listed in Table 6.

**Table 5:** Sequence of the primers used for molecular identification of *Aspergillus* section *Flavi* isolates.

Gene	Gene name	Length bp	Primers		Sequence (Nucleotides: 5′→3′)
			Forward	Reverse	
*ITS* (1–2)	18S ribosomal RNA gene partial sequence Internal Transcribed Spacer	565–613	ITS1		5′-GGAAGTAAAGTCGTAACAAGG
				ITS 2	5′-TTGGTCCGTGTTTCAAGACG
*ITS* (4–5)		300–330	ITS5		5′-GGAAGTAAAAGTCGTAACAAGG
				ITS 4	5′-TCCTCCGCTTATTGATATGC
*benA -*	β-tubulin	1125	β-tub 2a		5′-GGTAACCAAATCGGTGCTGCTTTC
				β-tub 2b	5′-ACCCTCAGTGTAGTGACCCTTGGC
*cmdA*	Calmodulin	543	Cmd5		5′-CCGAGTACAAGGAGGCCTTC-3′
				Cmd6	5′-CCGATAGAGGTCATAACGTGG-3′

**Table 6:** Nucleotide accession numbers for *benA* and *cmdA* of *A. flavus* strains with atypical morphology, *A. tamarii* and *A. minisclerotigenes* strains isolated from Lebanese spices.

Strain	Species	Beta-tubulin	Calmodulin
J4a	*Aspergillus tamarii*	MG957155	MG957162
J6a	*Aspergillus tamarii*	MG957156	MG957163
J31a	*Aspergillus tamarii*	MG957157	MG957164
J37	*Aspergillus tamarii*	MG957158	MG957165
J63c	*Aspergillus tamarii*	MG957159	MG957166
J116b	*Aspergillus tamarii*	MG957160	MG957167
J118a	*Aspergillus tamarii*	MG957161	MG957168
J117c	*Aspergillus minisclerotigenes*	MG957169	MG957170
J4c	*Aspergillus flavus*	MG957171	MG957174
J75e	*Aspergillus flavus*	MG957172	MG957175
J123a	*Aspergillus flavus*	MG957173	MG957176

#### Mycotoxigenic potential of isolates

To assess the toxigenic potential of *Aspergillus* section *Flavi* strains, a spore suspension was prepared from a 7-day culture, and 100 spores were centrally inoculated on MEA and cultured for seven days at 25 °C. After this incubation period, three sporulating cultures were analysed for toxin production. In parallel, at the end of the incubation period, three other cultures were used to perform a CFU count as previously described (see paragraph 4.3).

Toxins were then extracted from the whole culture medium by mechanical agitation in appropriate solvents. Extracts were filtered, and toxins were, as a first screening step, separated by thin layer chromatography (TLC) and quantified by fluorodensitometry using a Shimadzu CS930 fluorodensitometer (Shimadzu Corp., Kyoto, Japan), as previously described^9^. Results were further confirmed by HPLC analysis.

Evaporated extracts were diluted with 500 μL of water-acetonitrile-methanol (65:17.5:17.5, *v*/*v*/*v*) and filtered through a 0.45 μm disk (Thermo Scientific Fisher, Villebon-Sur-Yvette, France). Sample analysis was performed with a Dionex UltiMate 3000 UHPLC system (Thermo Scientific, Illkirch, France) using a liquid chromatography column Luna^®^ C18 (125 × 2 mm, 5 μm, 100 Å) (Phenomenex, Torrance, CA, USA) at 30 °C. Separation conditions were adapted from Fu *et al*.^49^, with slight modifications. Briefly, a 20 min isocratic mode program was delivered at 82.5% of eluent A: acidified water (0.2% of acetic acid) and acetonitrile (79:21, *v*/*v*) and 17.5% of eluent B: pure methanol. The flow rate was at 0.2 mL/min with a sample injection volume of 10 μL. Aflatoxins were detected using a fluorescent detector at 365/430 nm excitation/emission wavelengths. Peak identity was further confirmed by analysis of the absorption spectrum with a diode array detector (DAD) coupled to the system. Production levels of aflatoxins in media were calculated based on a standard calibration curve.

Based on their toxigenic potential (AFB+/−, AFG+/−, CPA+/−), isolates were classified into five chemotypes as proposed by Vaamonde *et al*.^50^: Chemotype I for AFB and CPA producers; Chemotype II for AFB, AFG and CPA producers; Chemotype III for AFB-only producers; Chemotype IV for CPA-only producers; and Chemotype V for non-toxigenic isolates.

## Supplementary information

Supplementary tables 1 and 2
