# Supplementary material for: Morphologic, molecular and metabolic characterization of Aspergillus section Flavi in spices marketed in Lebanon
Source: Sci Rep. 2019 Mar 27;9:5263. doi: 10.1038/s41598-019-41704-1 (PMC6437153; doi:10.1038/s41598-019-41704-1)
Supplement: Supplementary file 1 — Supplementary tables 1 and 2 [file 41598_2019_41704_MOESM1_ESM.pdf]

Morphologic, molecular and metabolic characterization of *Aspergillus* section *Flavi* in spices marketed in Lebanon

MAKHLOUF Joya[1,2], CARVAJAL-CAMPOS Amaranta [1], QUERIN Arlette [1], TADRIST Soraya [1], PUEL Olivier [1], LORBER Sophie [1], OSWALD Isabelle P[1], HAMZE Monzer [2], BAILLY Jean-Denis [1*], BAILLY Sylviane [1]

[1:] Toxalim (Research Center in Food Toxicology), Université de Toulouse, INRA, ENVT, INP-Purpan, UPS, 180 Chemin de Tournefeuille, F-31027, Toulouse, France.

[2:] Health and Environment Microbiology Laboratory, Lebanese University, Beirut, Lebanon.

* Corresponding author: jd.bailly@envt.fr

MAKHLOUF Joya, CARVAJAL-CAMPOS Amaranta, QUERIN Arlette, TADRIST Soraya, PUEL Olivier, LORBER Sophie, OSWALD Isabelle, HAMZE Monzer, BAILLY Jean-Denis , BAILLY Sylviane. Morphologic, molecular and metabolic characterization of Aspergillus section Flavi in spices marketed in Lebanon

Supplementary table 1: fungal analysis of spice samples from different regions of Lebanon

			Genus *Aspergillus*											Other fungal genera																
**Spices**	**Geographic origin**	**State**	*A.* section *aspergillus*	*A.* section *candidi*	*A.* section *circumdati*	***A.* section *flavi***	*A.* section *fumigati*	*A.* section *nidulantes*	*A.* section *nigri*	*A.* section *restricti*	*A.* section *terrei*	*A.* section *versicolores*	*A.* section *wentii*	*Absidia*	*Acremonium*	*Alternaria*	*Cladosporium*	*Fusarium*	*Hemispora*	*Mucor*	*Paecylomyces*	*Penicillium*	*Rhizomucor*	*Rhizopus*	*Spicaria*	*Syncephalastrum*	*Verticillium*	sterile *mycelia*	**total count (CFU/g)**	Yeast (/g)
Anise 1	Akkar	Powder	3,0E+01																					1,0E+01				1,0E+01	**5,0E+01**	1,0E+02
Anise 2	Borj Hammoud	Grain																						2,0E+02					**2,0E+02**	4,0E+04
Anise 3	Tripoli	Powder	1,0E+02						3,0E+02								1,0E+02	5,0E+02			5,0E+02	2,0E+02						1,0E+02	**1,8E+03**	1,0E+03
Anise 4	Bekaa	Powder				**3,0E+02**			3,0E+02								2,0E+02					1,0E+04						5,0E+02	**1,1E+04**	5,0E+03
Anise 5	Bekaa	Powder									2,0E+02																		**2,0E+02**	
Anise 6	Beyrouth	Powder																												2,0E+02
Bhar Al Kaak 1	Akkar	Powder	1,0E+02			**4,0E+01**			2,0E+02		1,0E+01	1,0E+01										1,0E+02						1,0E+02	**5,6E+02**	2,0E+03
Bhar Al Kaak 2	Beyrouth	Powder	3,0E+03		1,0E+02	**5,5E+02**		1,0E+01	1,0E+02			2,0E+01		1,0E+01								2,0E+02		3,0E+01		1,0E+01			**4,0E+03**	1,0E+03
Bhar Al Kaak 3	Zahle	Powder*	4,0E+03			**3,0E+01**			1,0E+02													2,0E+02		1,0E+02					**4,4E+03**	3,0E+03
Bhar Al Kaak 4	Baalbeck	Powder	1,0E+03			**1,0E+01**		2,0E+01	3,0E+02			4,0E+02		5,0E+01						5,0E+01		6,0E+01		1,0E+01					**1,9E+03**	3,0E+03
Bhar Al Kaak 5	Batroun	Powder*	7,0E+03			**2,0E+02**			2,0E+03			5,0E+01								6,0E+01				7,0E+02					**1,0E+04**	2,0E+03
Black pepper 1	Beyrouth	Powder	5,0E+03			**1,0E+01**			5,0E+01		1,0E+02	3,0E+02										2,0E+01		5,0E+01	1,0E+01				**5,5E+03**	
Black pepper 2	Tripoli	Powder	2,0E+03			**4,0E+03**			1,0E+03								1,0E+02					6,0E+03							**1,3E+04**	1,0E+03
Black pepper 3	Bekaa	Powder	1,5E+03	1,0E+02			5,0E+02		2,0E+02																				**2,3E+03**	1,0E+02
Black pepper 4	Borj Hammoud	Powder	1,0E+02	1,0E+02		**1,0E+02**		1,0E+02											3,0E+02			2,0E+02							**9,0E+02**	
Black pepper 5	Bekaa	Powder	1,0E+02	1,0E+02		**1,0E+02**											1,0E+02			1,0E+02								1,0E+02	**6,0E+02**	6,0E+03
Black pepper 6	Jnoub	Powder	3,0E+02				1,0E+02		1,0E+02																				**5,0E+02**	
Caraway 1	Tripoli	Powder	4,0E+02					2,0E+01	1,0E+02													2,0E+01						2,0E+01	**5,6E+02**	1,0E+02
Caraway 2	Borj Hammoud	Powder	1,0E+02						1,0E+02																		1,0E+02		**3,0E+02**	1,0E+02
Caraway 3	Hermel	Powder	3,0E+02						1,0E+03		1,0E+03						5,0E+02					1,0E+03							**3,8E+03**	2,0E+02
Caraway 4	Beyrouth	Powder	2,0E+03	1,0E+02																									**2,1E+03**	
Caraway 5	Koura	Powder*				**5,0E+03**			1,0E+02								1,0E+02				1,0E+02								**5,3E+03**	3,0E+03
Caraway 6	Jbeil	Powder*	2,0E+03																		1,0E+02							1,0E+03	**3,1E+03**	2,0E+03
Caraway 7	Jnoub	Powder*							1,0E+02																				**1,0E+02**	
Chili 1	Bcharre	Powder	3,0E+03			**1,1E+03**			2,0E+03															3,0E+02					**6,4E+03**	
Chili 2	Zahle	Powder*																						2,0E+02					**2,0E+02**	
Chili 3	Tripoli	Powder				**2,2E+03**												2,0E+03						1,0E+02					**4,3E+03**	2,0E+03
Chili 4	Hermel	Powder	2,0E+03			**1,0E+03**			5,0E+03		1,0E+03							1,0E+03				1,0E+03		1,0E+02					**1,1E+04**	
Chili 5	Beyrouth	Powder				**3,0E+03**			3,0E+04																				**3,3E+04**	
Chili 6	Baalbeck	Powder																										2,0E+02	**2,0E+02**	
Chili 7	Koura	Powder*				**3,0E+03**		4,0E+03	5,0E+04																				**5,7E+04**	6,0E+03
Cinnamon 1	Tripoli	Powder																											**0,0E+00**	
Cinnamon 2	Beyrouth	Powder																											**0,0E+00**	
Cinnamon 3	Baalbeck	Powder																				1,0E+02						2,0E+03	**2,1E+03**	5,0E+02
Cinnamon 4	Jbeil	Stick																											**0,0E+00**	
Cinnamon 5	Jnoub	Stick																											**0,0E+00**	
Coriander 1	Zgharta	Powder	2,0E+03																	2,0E+02		1,0E+01							**2,2E+03**	2,0E+02
Coriander 2	Borj Hammoud	Powder	5,0E+02						1,0E+02											1,0E+02									**7,0E+02**	5,0E+02
Coriander 3	Zahle	Powder	9,0E+02			**1,0E+02**	4,0E+02										1,0E+02				1,0E+02								**1,6E+03**	1,0E+02
Coriander 4	Hermel	Powder									3,0E+02																		**3,0E+02**	3,0E+02
Coriander 5	Tripoli	Powder	1,0E+02																									1,0E+02	**2,0E+02**	
Coriander 6	Beyrouth	Powder	1,0E+03			**4,0E+02**																							**1,4E+03**	
Cumin 1	Bekaa	Powder																4,0E+02											**4,0E+02**	3,0E+02
Cumin 2	Bekaa	Powder	1,0E+02																		1,0E+02	1,0E+02							**3,0E+02**	3,0E+02
Cumin 3	Tripoli	Powder				**1,0E+02**			2,0E+02						4,0E+02					1,0E+02									**8,0E+02**	5,0E+02
Cumin 4	Borj Hammoud	Powder							1,0E+02		2,0E+02						1,0E+02					2,0E+03		2,0E+02					**2,6E+03**	1,0E+02
Cumin 5	Beyrouth	Powder	5,0E+02				5,0E+02	3,0E+02								1,0E+03													**2,3E+03**	2,0E+02
Curry 1	Zgharta	Powder*	3,0E+04			**1,0E+05**		1,0E+03	3,0E+04			1,0E+03												1,0E+03					**1,6E+05**	1,0E+03
Curry 2	Tripoli	Powder	5,0E+03			**6,0E+02**			1,0E+03	2,0E+03	1,0E+02													1,0E+02					**8,8E+03**	1,0E+03
Curry 3	Jbeil	Powder	5,0E+02																					1,0E+02					**6,0E+02**	
Curry 4	Bekaa	Powder	4,0E+02	3,0E+02		**2,0E+02**			2,0E+02								1,0E+02										1,0E+02		**1,3E+03**	
Curry 5	Borj Hammoud	Powder							1,0E+02						2,0E+02									2,0E+02					**5,0E+02**	
Curry 6	Jnoub	Powder*																											**0,0E+00**	2,0E+02
Curry 7	Beyrouth	Powder				**4,0E+03**			3,0E+04															1,0E+02					**3,4E+04**	
Ginger 1	Zahle	Powder																											**0,0E+00**	
Ginger 2	Hermel	Powder	2,0E+02													2,0E+02		7,0E+02				2,0E+02							**1,3E+03**	2,0E+02
Ginger 3	Beyrouth	Powder																											**0,0E+00**	
Ginger 4	Koura	Powder*		6,0E+03													1,0E+03					3,0E+04							**3,7E+04**	
Ginger 5	Jnoub	Powder*	7,0E+03																										**7,0E+03**	

Lebanese pepper 1	Zgharta	Powder	5,0E+03			**8,0E+02**	7,0E+03		1,0E+03												3,0E+03	5,0E+01							**1,7E+04**	
Lebanese pepper 2	Beyrouth	Powder	6,0E+02				2,0E+01							1,0E+01								5,0E+01							**6,8E+02**	
Lebanese pepper 3	Baalbeck	Powder	1,0E+03		1,0E+01				1,0E+02					2,0E+01							1,0E+02	1,0E+01	2,0E+01						**1,3E+03**	3,0E+01
Lebanese pepper 4	Mont Liban	Powder*	1,0E+03						1,0E+02					1,0E+02						1,0E+02									**1,3E+03**	
Lebanese pepper 5	Al Koura	Powder*	7,0E+04	1,0E+01		**2,1E+02**	2,0E+04	1,0E+02	5,0E+03		1,0E+01	1,0E+03	1,0E+02									1,0E+02							**9,7E+04**	
Nutmeg 1	Tripoli	Powder	3,0E+03											2,0E+01						1,0E+01									**3,0E+03**	
Nutmeg 2	Borj Hammoud	Powder	6,0E+02						1,0E+03	2,0E+02																			**1,8E+03**	
Nutmeg 3	Jnoub	Powder	1,0E+03			**1,0E+03**	2,0E+02		2,0E+02						1,0E+02														**2,5E+03**	
Nutmeg 4	Beyrouth	Nut	5,0E+03			**1,0E+03**																							**6,0E+03**	
Nutmeg 5	Baalbeck	Powder*					5,0E+02												1,0E+02		1,0E+02	6,0E+02						2,0E+02	**1,5E+03**	2,0E+03
Nutmeg 6	Koura	Powder*	1,0E+03			**1,0E+03**			6,0E+02																			7,0E+02	**3,3E+03**	2,0E+03
Turmeric 1	Tripoli	Powder																3,0E+02											**3,0E+02**	
Turmeric 2	Beyrouth	Powder				**1,0E+03**			1,0E+03		2,0E+03										1,0E+03							1,0E+02	**5,1E+03**	
Turmeric 3	Mont Liban	Powder*																						1,0E+02					**1,0E+02**	
Turmeric 4	Zahle	Powder*																											**0,0E+00**	
Turmeric 5	Beyrouth	Powder	5,0E+02	3,0E+02		**5,0E+02**			2,0E+01			3,0E+02										1,0E+02		5,0E+01					**1,8E+03**	
White pepper 1	Bekaa	Powder																											**0,0E+00**	
White pepper 2	Tripoli	Powder	4,0E+02			**1,0E+03**	5,0E+02		1,0E+02															1,0E+02					**2,1E+03**	2,0E+02
White pepper 3	Bekaa	Powder	2,0E+02																					1,0E+02					**3,0E+02**	2,0E+02
White pepper 4	Borj Hammoud	Powder																											**0,0E+00**	
White pepper 5	Beyrouth	Powder																											**0,0E+00**	

*: samples that were conditionned before analysis

MAKHLOUF Joya, CARVAJAL-CAMPOS Amaranta, QUERIN Arlette, TADRIST Soraya, PUEL Olivier, LORBER Sophie, OSWALD Isabelle, HAMZE Monzer, BAILLY Jean-Denis , BAILLY Sylviane.
Morphologic, molecular and metabolic characterization of *Aspergillus* section *Flavi* in spices marketed in Lebanon

Supplementary Table 2

Identification, origin, initial count and toxigenic potential of the 53 strains of *Aspergillus* section *Flavi* isolated from spices samples

Strain number	Species	Spice	Count in sample (CFU/g)	AFPA	Mycotoxin production profil (µg/PD)					Chemotypes	Sclerotia
					CPA	AFB1	AFB2	AFG1	AFG2		
**J58b**	*Aspergillus flavus*	Anise	100	+++	< LOQ	ND	ND	ND	ND	IV	-
**J58c**	*Aspergillus flavus*		200	(+)	57.5	ND	ND	ND	ND	IV	-
**J2**	*Aspergillus flavus*	Bhar el Kaak	40	++	172.1	146.4	< LOQ	ND	ND	I	-
**J117a**	*Aspergillus flavus*	Bhar al Kaak	300	+++	49.8	ND	ND	ND	ND	IV	Some big sclerotia (>>500 µm)
**J117b**	*Aspergillus flavus*		150	++	4532.5	335.9	< LOQ	ND	ND	I	-
**J117c**	*Aspergillus minisclerotigenes*		100	++	1216.48	26.9	ND	9.8	ND	II	Numerous little sclerotia (<500 µm)
**J118a**	*Aspergillus tamarii*	Bhar al Kaak	10	brown	487.2	ND	ND	ND	ND	IV	-
**J118b**	*Aspergillus flavus*		20	++	41.5	ND	ND	ND	ND	IV	-
**J119**	*Aspergillus flavus*	Bhar al Kaak	10	+++	2635.2	77.1	< LOQ	ND	ND	I	-
**J120**	*Aspergillus flavus*	Bhar al Kaak	200	++	1534.2	<LOQ	ND	ND	ND	I	-
**J8**	*Aspergillus flavus*	Black pepper	10	++	ND	<LOQ	ND	ND	ND	III	-
**J31**	*Aspergillus flavus*	Black pepper	200	+++	24.7	ND	< LOQ	ND	ND	I	-
**J31a**	*Aspergillus tamarii*		1000	brown	<LOQ	ND	ND	ND	ND	IV	-
**J31b**	*Aspergillus flavus*		800	++	46.2	ND	ND	ND	ND	IV	-
**J31c**	*Aspergillus flavus*		2000	++	108.7	172,8	ND	ND	ND	I	-
**J33a**	*Aspergillus flavus*	Black pepper	100	+	ND	ND	ND	ND	ND	V	-
**J34a**	*Aspergillus flavus*	Black pepper	100	+++	ND	ND	ND	ND	ND	V	-
**J85**	*Aspergillus flavus*	Caraway	5000	+++	ND	ND	ND	ND	ND	V	-
**J4a**	*Aspergillus tamarii*	Chili	20	brown	389.3	ND	ND	ND	ND	IV	-
**J4b**	*Aspergillus flavus*		50	++	376.5	213.5	< LOQ	ND	ND	I	-
**J4c**	*Asperglllus flavus*		1000	(+)	ND	<LOQ	ND	ND	ND	III	-
**J62a**	*Aspergillus flavus*	Chili	2000	++	<LOQ	ND	ND	ND	ND	IV	-
**J62b**	*Aspergillus flavus*		200	++	<LOQ	ND	ND	ND	ND	IV	-
**J63a**	*Aspergillus flavus*	Chili	300	++	100.8	134	ND	ND	ND	I	-
**J63c**	*Aspergillus tamarii*		400	brown	161.6	ND	ND	ND	ND	**IV**	-
**J63d**	*Aspergillus flavus*		300	+++	72.3	0.5	ND	ND	ND	I	-
**J71a**	*Aspergillus flavus*	Chili	1000	++	332.7	251.5	ND	ND	ND	I	-
**J71b**	*Aspergillus flavus*		2000	++	1148.4	193.1	< LOQ	ND	ND	I	-
**J84a**	*Aspergillus flavus*	Chili	2000	++	ND	145.3	ND	ND	ND	III	-
**J84b**	*Aspergillus flavus*		1000	++	ND	ND	ND	ND	ND	V	-
**J53b**	*Aspergillus flavus*	Coriander	100	++	76,7	ND	ND	ND	ND	IV	-
**J76a**	*Aspergillus flavus*	Coriander	200	++	<LOQ	ND	ND	ND	ND	IV	-
**J76b**	*Aspergillus flavus*		200	(+)	<LOQ	ND	ND	ND	ND	IV	-
**J50**	*Aspergillus flavus*	Cumin	100	(+)	ND	ND	ND	ND	ND	V	-
**J6a**	*Aspergillus tamarii*	Curry	500	brown	327.2	ND	ND	ND	ND	IV	-
**J6b**	*Aspergillus flavus*		100000	++	199.5	318.5	< LOQ	ND	ND	I	-
**J42a**	*Aspergillus flavus*	Curry	400	++	ND	ND	ND	ND	ND	V	-
**J42b**	*Aspergillus flavus*		200	++	215.5	0.3	ND	ND	ND	I	-
**J44a**	*Aspergillus flavus*	Curry	200	++	ND	ND	ND	ND	ND	V	-
**J69a**	*Aspergillus flavus*	Curry	4000	++	243	76.5	ND	ND	ND	I	-
**J7**	*Aspergillus flavus*	Lebanese pepper	800	++	455.5	ND	ND	ND	ND	IV	Numerous small sclerotia (±500 µm)
**J116a**	*Aspergillus flavus*	Lebanese pepper	200	++	2607.3	30.1	< LOQ	ND	ND	I	-
**J116b**	*Aspergillus tamarii*		10	Brown	1226	ND	ND	ND	ND	IV	-
**J68a**	*Aspergillus flavus*	Nutmeg	1000	+++	ND	ND	ND	ND	ND	V	-
**J70**	*Aspergillus flavus*	Nutmeg	1000	++	ND	53,1	ND	ND	ND	III	-
**J86a**	*Aspergillus flavus*	Nutmeg	1000	+	126.8	578.4	ND	ND	ND	I	Some big sclerotia (>500 µm)
**J75a**	*Aspergillus flavus*	Turmeric	500	+	ND	148.6	ND	ND	ND	III	-
**J75c**	*Aspergillus flavus*		200	+	ND	351.1	ND	ND	ND	III	-
**J75g**	*Aspergillus flavus*		100	+	125.4	124.6	ND	ND	ND	I	-
**J75e**	*Aspergillus flavus*		200	+	252.5	18.2	ND	ND	ND	I	-
**J123a**	*Aspergillus flavus*	Turmeric	200	+++	ND	ND	ND	ND	ND	V	-
**J123b**	*Aspergillus flavus*		300	++	388.1	ND	ND	ND	ND	IV	-
**J37**	*Aspergillus tamarii*	White pepper	1000	Brown	75.2	ND	ND	ND	ND	IV	-

PD: Petri Dish

CFU: colon y forming unit; AFPA: Aspergillus flavus and parasiticus Agar; CPA: cyclopiazonic acid

Chemotype I: AFB+/CPA+; Chemotype II: AFB+/ AFG+//CPA+; Chemotype III: AFB+; Chemotype IV: CPA+; Chemotype V: non-toxigenic

ND: Not detected (< Limit of detection)

LOQ: limit of quantification
